# What doctors think about the impact of managed care tools on quality of care, costs, autonomy, and relations with patients

**DOI:** 10.1186/1472-6963-10-331

**Published:** 2010-12-07

**Authors:** Marie Deom, Thomas Agoritsas, Patrick A Bovier, Thomas V Perneger

**Affiliations:** 1 Division of Clinical Epidemiology, University Hospitals of Geneva, and School of Medicine, University of Geneva, Geneva, Switzerland

## Abstract

**Background:**

How doctors perceive managed care tools and incentives is not well known. We assessed doctors' opinions about the expected impact of eight managed care tools on quality of care, control of health care costs, professional autonomy and relations with patients.

**Methods:**

Mail survey of doctors (N = 1546) in Geneva, Switzerland. Respondents were asked to rate the impact of 8 managed care tools on 4 aspects of care on a 5-level scale (1 very negative, 2 rather negative, 3 neutral, 4 rather positive, 5 very positive). For each tool, we obtained a mean score from the 4 separate impacts.

**Results:**

Doctors had predominantly negative opinions of the impact of managed care tools: use of guidelines (mean score 3.18), gate-keeping (2.76), managed care networks (2.77), second opinion requirement (2.65), pay for performance (1.90), pay by salary (2.24), selective contracting (1.56), and pre-approval of expensive treatments (1.77). Estimated impacts on cost control were positive or neutral for most tools, but impacts on professional autonomy were predominantly negative. Primary care doctors held more positive opinions than doctors in other specialties, and psychiatrists were in general the most critical. Older doctors had more negative opinions, as well as those in private practice.

**Conclusions:**

Doctors perceived most managed care tools to have a positive impact on the control of health care costs but a negative impact on medical practice. Tools that are controlled by the profession were better accepted than those that are imposed by payers.

## Background

"Managed care" is a global term for health care systems that integrate the delivery and financing of health care. Managed care contrasts with liberal medical practice, which allows doctors to make clinical decisions and bill for their services without interference from managers or payers. Traditional forms of managed care include the staff-model health maintenance organization (HMO) and the office-based independent provider association [1,2]. However, many variants exist. Luft notes that "in reality, each HMO is a highly complex combination of economic incentives, bureaucratic structures, and personalities" [3]. Another definition characterizes managed care programs by their use of a variety of interventions, including economic incentives for doctors and patients, review of the medical necessity of specific services, increased cost sharing, controls on inpatient admissions and lengths of stay, and selective contracting with health care providers [4].

Thus managed care is akin to a tool-box. The tools include the use of practice guidelines [5], gate-keeping [6], health care networks [7], second opinion requirements [8], and pre-approval requirements for expensive treatments or hospitalisation [9,10] (or utilization review). Other tools act through financial incentives: doctor payment for performance or by salary instead of fee-for-service payment [11,12], as well as the possibility for insurers to choose which doctors to reimburse (selective contracting) [13].

While managed care originated in North America, its tools have spread internationally. For instance, generalists in several Northern European countries regulate access to specialists, and have responsibility over a per capita annual budget. France has created a governmental agency to develop clinical practice guidelines. In Switzerland, the Health Insurance Law of 1996 has authorised managed care plans that apply various cost containment measures [14]. Therefore managed care tools have become a concern for doctors in many countries.

How doctors perceive these tools is not well known. Several studies have explored doctors' perceptions of practice guidelines and managed care in general [7,15-22], but evidence is scant concerning other tools [23-26]. Because the methods and survey instruments vary across studies, direct comparisons of various tools are impossible. Furthermore, global opinions such as overall acceptability may hide differences in specific impacts. For example, doctors may believe that utilization review will reduce total health care expenditures but that quality of care and access to treatments may suffer. In addition to quality and costs, managed care tools may also have an impact on doctors' autonomy and on their relationships with patients. We are not aware of studies comparing various managed care tools on their various perceived impacts. Understanding doctors' perceptions of managed care tools is important, because the doctors' acceptance of such tools is required for their successful implementation.

The aim of this study was to explore doctors' opinions about the impact of eight managed care tools (use of guidelines, gate-keeping, health care networks, second opinion requirement, doctor payment by performance or salary, selective contracting, and pre-approval of expensive treatments) on four aspects of medical care (quality of care, cost control, professional autonomy and relations with patients).

## Method

### Setting

This study was conducted in canton Geneva, Switzerland. The Swiss health care system mixes liberal medical practice in ambulatory care with a publicly run hospital sector. Managed care, as implemented in the United States, is not much developed; about <10% of the population is enrolled in managed care plans. Reinhardt describes Swiss health care as a "consumer directed health care" system with tight government rules [14]. The majority of hospitals are public and most hospital doctors are paid by salary (only a few senior doctors are allowed to have a part-time private practice at the hospital). Doctors in private practice are reimbursed on the basis of a fee-for-service schedule negotiated between caregivers and insurers. Swiss residents are covered by mandatory health insurance; most have access to any doctor of their choice, and only a fraction are enrolled in managed care networks. Health insurance premiums vary across regions (cantons) and insurance carriers but are identical within an insurance company and canton for all adults aged 25 or older (children and young adults pay lower premiums), regardless of health status or pre-existing conditions. Premiums can be lowered by choosing a higher annual deductible (up to 2500 Swiss francs) or managed care plans. The government subsidizes low-income residents, and also determines which drugs and services are reimbursed under the basic health insurance plan. In addition to basic insurance, some people contract private insurance policies to reimburse additional services, mostly hospitalization in private clinics. Health care expenditures represented 10.6% of the gross domestic product in 2007 and were shared between households (31.7%), private insurance (9.2%), the state (16.2%), and social insurance (42.9%) [27].

Managed care is a relevant topic for Swiss doctors. In addition to the possibility to join existing managed care plans, two measures are currently discussed: selective contracting is promoted by the health insurers, and managed care networks, coordinated by primary care doctors, are promoted in federal policy circles.

### Study design and sample

We conducted a mail survey of all doctors active in clinical care in Geneva, Switzerland. We included doctors who worked at public hospitals or in private practice, and who were board certified or still in training. We used two databases: the Geneva Medical Association membership register and the University Hospitals of Geneva human resources file. After exclusion of duplicate records, invalid addresses and doctors who did not work with patients (e.g., pathologists, public health specialists, etc), 2746 doctors remained eligible. Our sample matched the number of doctors in Geneva according to the Swiss Medical Association [28]. We sent to each doctor the initial survey package and up to two reminder packages between November 2007 and February 2008. This study was approved by the Research Ethics Committee of the University Hospitals of Geneva.

### Attitudes toward management tools

This section of the questionnaire started with the following statement: "In recent times, new measures have changed the liberal practice of medicine, and several others are being discussed. Please check for each of the following measures if you judge that it has or would have a positive or negative impact on professional autonomy, cost control, quality of care, and relations with patients." This was followed by a list of eight managed care tools: (a) systematic use of guidelines for clinical practice (abbreviated as "guidelines"), (b) patients' access to specialists guided by a primary care doctor (gatekeeping), (c) necessity to ask the insurance carrier for an authorization before any hospitalisation (utilization review), (d) necessity to ask for a second medical opinion for any costly investigation or treatment (second opinion), (e) doctors' participation in health care networks which coordinate care for affiliated patients (network), (f) possibility for the insurer to choose which doctor he will reimburse (selective contracting), (g) doctor's pay for performance based on cost control, patient satisfaction, or quality of care (pay for performance), and (h) payment by salary (salary). Each managed care tool was followed by a list of four impacts - professional autonomy, control of health care costs, quality of care, and relations with patients. Possible answers to each item were: 1 = very negative, 2 = rather negative, 3 = neutral, 4 = rather positive and 5 = very positive. There were 32 assessments in total (4 impacts for 8 tools).

The questions were developed by the investigators, and were pretested in face to face interviews with 15 doctors.

### Other variables

The questionnaire included questions on the respondent's sex, year of birth, specialty (either completed or planned) and practice setting (private practice in solo, in group or in a clinic or salaried practice in a clinic or a public hospital). Respondents were also asked where and when they graduated, if and when they received their specialty certification, and whether they were part of any managed care network.

The questionnaire also evaluated work satisfaction and opinions on insurance policies and quality of care; these topics are not discussed in this article.

### Statistical analysis

We described the response rate and the characteristics of participants, comparing respondents and non-respondents. As negative opinions globally predominated, we reported proportions of negative opinions regarding each tool's impact on each aspect (autonomy, costs, quality, and relations with patients), grouping "very negative" and "rather negative" opinions.

The four impact ratings turned out to be correlated, for all of the managed care tools. Therefore we constructed global opinion scores for each of the 8 tools as the average of the 4 expected impacts. For each scale, we reported the internal consistency coefficient (Cronbach alpha). However, absolute expected impacts (i.e., negative or positive) differed across the four domains for most managed care tools, so descriptive statistics are shown separately for each of the 32 ratings (4 impacts for 8 tools).

Scores were compared across subgroups of respondents (sex, age, specialty, practice setting and membership in a managed care network). We classified specialties into five groups: primary care doctors (generalists and general internists), internal medicine specialists (including neurologists), paediatricians, psychiatrists and technical specialists (surgeons, anaesthetists, ear-nose-throat specialists, ophthalmologists, dermatologists, gynaecologists-obstetricians and radiologists). We distinguished between three categories of practice settings: independent private practice, public hospital practice in training or public hospital practice as senior.

For each tool impact score we built a multivariate model using doctor characteristics as predictors. We used SPSS 15.0 for all analyses.

## Results

### Sample characteristics

The survey response rate was of 56.3% (1546/2746). Participation was not related to age, setting of practice and source data base. However men responded more frequently than women (58.0% vs. 53.7%, p = 0.027) and participation varied according to specialty, from 52.6% in technical specialists to 62.2% in primary care doctors (p = 0.003).

The sample included more men than women (Table 1). More than half of all respondents worked as independent doctors, either in solo or in group practice and one out of five belonged to a managed care network. Primary care doctors and technical specialists represented each about a third of the sample, the remaining third consisting of psychiatrists, internal medicine specialists and paediatricians. On average, doctors were 47.3 years old (SD 11.6) and graduated 20.1 years ago (SD 11.1). Specialists had obtained their certification 14.0 years ago (SD 9.4) on average, and those in private practice established their practice 15.2 years ago (SD 9.0).

**Table 1:** Characteristics of doctors who participated in the opinion survey regarding managed care tools (N = 1546), Geneva, Switzerland, 2007.

Characteristics	N (%) ^#^
Sex	
men	956 (61.9)
women	589 (38.1)

Age in years	
up to 35	304 (19.7)
36-50	612 (39.6)
over 50	628 (40.7)

Practice setting	
private practice	877 (56.7)
public in training	515 (33.3)
public senior	154 (10.0)

Specialty	
primary care doctors	445 (28.9)
paediatricians	128 (8.3)
psychiatrists	286 (18.6)
technical specialists	449 (29.2)
internal medicine specialists	231 (15.0)

Participation in managed care network*	
yes	173 (19.9)
no	695 (80.1)

* Among private practitioners (N = 868)

^# ^Total may differ from 1546 because of missing values ranging from 0 to 7 (0-0.45%).

Most doctors who worked in managed care belonged to one or two of 4 organizations: A (N = 88), B (N = 87), and C (N = 25), and D (N = 18). Organisations A and B were independent provider organisations that included generalists and internal medicine specialists; both had a program of quality circles, practiced gate-keeping (gynaecology check-ups and paediatric consultations were not subjected to gate-keeping), and used fee-for-service payments. Neither used direct financial incentives or imposed a global budget, and both had contracts with several health insurance carriers. Organisation C was similar, but was restricted to care for migrants and asylum seekers, and worked more closely with the public hospital in providing care to this population. Organization D was a group practice set up by a single insurer, where doctors were paid by salary. None of the participants had been exposed to pay for performance, utilisation review, or selective contracting.

### Proportions of negative opinions

Use of guidelines received the smallest proportion of negative opinions (18.0% to 35.6%) followed by gate-keeping and health care networks (Table 2; full item distributions of the items are available from the last author on request). In contrast, pay for performance, utilization review and selective contracting gathered the highest percentages of negative judgments. Payment by salary and second opinion stood in between. Respondents reported more often negative impacts on professional autonomy and relations with patients than on quality of care and on the control of health care costs.

**Table 2:** Absolute numbers and proportions of negative perceptions for each managed care tool and its impacts*, in 1546 doctors in Geneva, Switzerland, 2007.

	Very negative or rather negative impact on			
	
	Professional autonomy N (%)	Relation with patients N (%)	Quality of care N (%)	Control of health care costs N (%)
Use of guidelines	536 (35.6)	437 (29.0)	293 (19.5)	269 (18.0)

Gate-keeping	938 (61.9)	675 (44.6)	569 (37.7)	419 (27.8)

Managed care network	908 (60.3)	626 (41.7)	479 (31.9)	301 (20.1)

Second opinion	1024 (67.6)	803 (53.0)	458 (30.3)	547 (36.1)

Pay by salary	1009 (66.8)	856 (56.8)	943 (62.7)	618 (41.6)

Pay for performance	1326 (87.9)	1228 (81.7)	1162 (77.3)	721 (48.4)

Utilization review	1448 (95.3)	1352 (89.1)	1268 (83.7)	713 (47.4)

Selective contracting	1475 (96.8)	1419 (93.2)	1398 (91.9)	837 (55.4)

* Percentages of valid responses (maximum missing is 61)

### Global impact scores

Cronbach alpha coefficients for impacts of the four aspects of care within each tool ranged from 0.75 to 0.89 (Table 3). Mean scores ranged from 1.56 (selective contracting) to 3.18 (guidelines), and standard deviations were all less than 1. Use of guidelines was the only tool with an average score above 3, slightly better than neutral. Gate-keeping, health care networks, second opinion and payment by salary were rated between 2 and 3 on average (between neutral and rather negative). Pay for performance, utilization review and selective contracting, had mean scores below 2 (between rather and very negative).

**Table 3:** Global scores for each tool, computed as the average of the four impact-specific items, among 1546 doctors in Geneva, Switzerland, 2007.

	Number of items	Cronbach alpha coefficient	Mean (SD)*
Use of guidelines	4	0.83	3.18 (0.80)
Gatekeeping	4	0.87	2.76 (0.89)
Managed care network	4	0.89	2.77 (0.81)
Second opinion	4	0.83	2.65 (0.81)
Pay by salary	4	0.89	2.24 (0.93)
Pay for performance	4	0.87	1.90 (0.81)
Utilization review	4	0.76	1.77 (0.59)
Selective contracting	4	0.75	1.56 (0.58)

* Higher = more positive on a scale from 1 to 5

### Differences across subgroups

Mean global impact scores for each tool varied across subgroups of respondents in univariate (Table 4) and multivariate analysis (Table 5). Men had a more positive attitude than women concerning second opinion and pay for performance, but rated payment by salary more negatively. These differences decreased in multivariate analysis. Older respondents reported more negative opinions about all tools except about second opinion. In multivariate analysis, age differences remained significant only for guidelines, gate-keeping and second opinion.

**Table 4:** Univariate analysis, Mean score (SD) of each tool across subgroups of respondents, in 1546 doctors in Geneva, Switzerland, 2007.

	Use of guidelines (N = 1491)	Gate-keeping (N = 1499)	Managed care network (N = 1490)	Second opinion (N = 1506)	Pay by salary (N = 1480)	Pay for performance (N = 1487)	Utilization review (N = 1500)	Selective contracting (N = 1506)
Sex	*p = 0.91*	*p = 0.63*	*p = 0.79*	*p = 0.001*	*P = 0.001*	*p = 0.004*	*p = 0.83*	*p = 0.55*
men	3.18 (0.80)	2.75 (0.91)	2.77 (0.82)	2.70 (0.81)	2.18 (0.93)	1.95 (0.82)	1.77 (0.59)	1.57 (0.59)
women	3.19 (0.81)	2.77 (0.86)	2.76 (0.79)	2.56 (0.81)	2.34 (0.93)	1.82 (0.78)	1.78 (0.58)	1.55 (0.58)

Age	*p < 0.001*	*p < 0.001*	*p < 0.001*	*p < 0.001*	*p < 0.001*	*p = 0.023*	*p = 0.29*	*p < 0.001*
up to 35	3.37 (0.65)	3.07 (0.77)	2.99 (0.72)	2.53 (0.75)	2.71 (0.87)	2.01 (0.85)	1.82 (0.55)	1.71 (0.64)
36-50	3.28 (0.76)	2.79 (0.86)	2.80 (0.81)	2.60 (0.78)	2.29 (0.95)	1.88 (0.79)	1.76 (0.58)	1.54 (0.56)
over 50	2.99 (0.87)	2.57 (0.92)	2.62 (0.82)	2.76 (0.86)	1.96 (0.84)	1.86 (0.80)	1.77 (0.61)	1.52 (0.56)

Practice setting	*p < 0.001*	*p < 0.001*	*p < 0.001*	*p = 0.016*	*p < 0.001*	*p < 0.001*	*p = 0.002*	*p < 0.001*
private practice	3.02 (0.83)	2.59 (0.93)	2.59 (0.79)	2.67 (0.85)	1.88 (0.80)	1.78 (0.75)	1.74 (0.59)	1.46 (0.50)
public in training	3.38 (0.70)	3.03 (0.78)	2.99 (0.76)	2.57 (0.75)	2.73 (0.89)	2.01 (0.84)	1.80 (0.57)	1.68 (0.63)
public senior	3.44 (0.75)	2.78 (0.74)	3.03 (0.79)	2.77 (0.80)	2.60 (0.85)	2.22 (0.86)	1.91 (0.59)	1.76 (0.71)

Specialty	*p < 0.001*	*p < 0.001*	*p < 0.001*	*p = 0.004*	*p < 0.001*	*p = 0.026*	*p < 0.001*	*p = 0.004*
primary care	3.28 (0.78)	3.25 (0.76)	2.98 (0.77)	2.70 (0.80)	2.36 (0.92)	1.87 (0.76)	1.72 (0.53)	1.59 (0.56)
paediatricians	3.36 (0.71)	2.89 (0.85)	2.75 (0.78)	2.53 (0.81)	2.32 (0.91)	1.87 (0.79)	1.71 (0.53)	1.52 (0.58)
psychiatrists	2.86 (0.83)	2.40 (0.81)	2.65 (0.78)	2.77 (0.81)	2.22 (0.90)	1.81 (0.76)	1.75 (0.56)	1.46 (0.48)
technical specialists	3.19 (0.81)	2.53 (0.87)	2.64 (0.81)	2.59 (0.83)	2.08 (0.94)	2.00 (0.91)	1.88 (0.65)	1.62 (0.68)
internal medicine specialists	3.27 (0.75)	2.60 (0.85)	2.71 (0.82)	2.57 (0.80)	2.26 (0.95)	1.91 (0.76)	1.75 (0.58)	1.54 (0.51)

Managed care network*	*p = 0.49*	*p < 0.001*	*p < 0.001*	*p = 0.015*	*p = 0.002*	*p = 0.39*	*p = 0.19*	*p = 0.42*
yes	3.15 (0.71)	3.29 (0.72)	3.14 (0.68)	2.79 (0.76)	2.04 (0.83)	1.85 (0.73)	1.72 (0.59)	1.53 (0.51)
no	3.19 (0.81)	2.69 (0.88)	2.72 (0.81)	2.63 (0.82)	2.27 (0.94)	1.91 (0.82)	1.78 (0.59)	1.57 (0.59)

*among private practitioners

**Table 5:** Differences in scores across subgroups of respondents, adjusted for all variables in the table.

	Use of guidelines	Gate-keeping	Managed care network	Second opinion	Pay by salary	Pay for performance	Utilization review	Selective contracting
Sex	*(p = 0.44)*	*(p = 0.06)*	*(p = 0.10)*	*(p = 0.003)*	*(p = 0.36)*	*(p = 0.006)*	*(p = 0.46)*	*(p = 0.48)*
women (vs men)	-0.03	-0.08	-0.07	-0.14	0.04	-0.12	0.02	-0.02

Age (vs >50 years)	*(p = 0.005)*	*(p = 0.11)*	*(p = 0.40)*	*(p = 0.003)*	*(p = 0.93)*	*(p = 0.46)*	*(p = 0.51)*	*(p = 0.11)*
up to 35	0.13	0.15	0.08	-0.27	-0.02	-0.06	0.03	0.03
36-50	0.17	0.10	0.07	-0.17	-0.02	-0.07	-0.03	-0.06

Practice setting (vs private practice)	*(p < 0.001)*	*(p < 0.001)*	*(p < 0.001)*	*(p = 0.05)*	*(p < 0.001)*	*(p < 0.001)*	*(p = 0.005)*	*(p < 0.001)*
public in training	0.30	0.48	0.49	0.09	0.88	0.31	0.04	0.21
public senior	0.37	0.33	0.58	0.17	0.79	0.46	0.18	0.32

Specialty (vs primary care)	*(p < 0.001)*	*(p < 0.001)*	*(p = 0.006)*	*(p = 0.002)*	*(p < 0.001)*	*(p = 0.20)*	*(p = 0.001)*	*(p = 0.04)*
paediatricians	0.05	-0.23	-0.09	-0.11	-0.08	0.02	-0.01	-0.07
psychiatrists	-0.40	-0.69	-0.14	0.08	-0.14	-0.03	0.05	-0.11
technical specialists	-0.11	-0.60	-0.20	-0.11	-0.34	0.11	0.17	0.02
internal medicine specialists	-0.02	-0.52	-0.17	-0.18	-0.10	0.01	0.03	-0.05

Managed care network*	*(p = 0.43)*	*(p < 0.001)*	*(p < 0.001)*	*(p = 0.27)*	*(p = 0.53)*	*(p = 0.16)*	*(p = 0.48)*	*(p = 0.33)*
yes (vs no)	0.06	0.50	0.58	0.09	0.05	0.11	0.04	0.05

* "no" was imputed for doctors at the public hospital

Most tools were rated lower by private practitioners than by doctors working in public hospitals. Practice differences persisted for almost every measure in multivariate analysis, except for second opinion and the utilization review.

Primary care doctors had a more positive attitude not only toward guidelines but also toward gate-keeping. Along with paediatricians, they had the highest scores for all tools except pay for performance, utilization review and selective contracting. For the latter, technical specialists held the most positive opinions. Psychiatrists appeared to be the most reluctant specialists, particularly concerning gate-keeping. Internal medicine specialists usually gave intermediate ratings. In multivariate analysis, differences between specialists remained strongest for ratings of pay for performance, utilization review, and selective contracting. Among independent practitioners, those involved in a medical network rated second opinion better than other doctors and also had a positive attitude toward gate-keeping and networks.

## Discussion

Overall, the Swiss doctors who participated in this study expressed negative opinions about the expected impact of most managed care tools. Only the use of guidelines received a lukewarm endorsement from the respondents. Perhaps predictably, managed care tools that remain under the control of the medical profession - guidelines, gate-keeping and health care networks - were rated more favourably than tools that put payers in charge, such as pay for performance, utilization review and selective contracting. These findings are compatible with previous observations from the United States that managed care reduces career satisfaction through its impact on doctor's autonomy [29], and that managed care constraints may hamper the doctor's ability to provide high quality care [30]. However, as we did not obtain the doctors' explanations of why they gave a positive or negative impact rating, the specific reasons for these opinions of Geneva doctors remain unknown. That doctor's attitudes are context-specific is illustrated in qualitative study comparing doctor's perceptions of practice guidelines in Norway and Denmark [31].

### Subgroup comparisons

Differences between specialties were rather small, with few exceptions. One was the more positive view of gatekeeping and networks by primary care doctors. It is understandable that generalists should support tools that give them an active role in the health care system. Other studies have suggested likewise [7,32]. In addition, general practitioners may be less motivated by financial incentives than other specialties [33], and therefore may be more willing to accept payers' intrusion into medical practice. Primary care doctors had opinions of guidelines that were similar to other specialities (excepting psychiatrists). Some previous studies have observed that generalists were less prone to using guidelines than specialists [34], but that was not the case in our data. The higher than average survey participation rate of primary care doctors' suggests that this group may be particularly interested in managed care issues.

For most tools, psychiatrists held more negative opinions than other doctors. Psychiatrists may feel particularly threatened by managed care [35], because traditional psychotherapy requires multiple doctor visits that may not be approved under many cost cutting policies.

Older doctors and those in private practice had more negative opinions of the various managed care tools. These are subgroups that have the greatest experience of a traditional "liberal" model of medical practice, grounded in professional autonomy and fee-for-service compensation, which is under pressure from managed care. Similar findings have been reported in previous studies of managed care [7] and guideline acceptability [15,17,21,22,33,36].

Private practitioners who were members of a managed care network had a more positive opinion of gate-keeping and health care networks, and salaried doctors rated payment by salary higher than other respondents. Whether this reflects a selection bias or the effect of greater familiarity with these measures is unclear. Previous research supports the notion that familiarity with some managed care tools is associated with more positive opinions [7,33,37]. If indeed familiarity led to positive opinions, implementation of managed care tools despite initial reluctance on the doctors' part may be a viable strategy. However, familiarity may improve perceptions only if *a priori *apprehensions are disproved by experience.

### Impact on different aspects of care

We have conducted most analyses using a summary score, the global impact score, for each managed care tool. This was justified by the high internal consistency coefficients of the summary scales, and allowed a synthetic presentation of the results. However, as others have reported [7,23], doctors differentiate the impact on costs from impacts on other aspects of care. Many doctors consider that these tools help rein in health expenditures, but few believe that they promote professional autonomy, quality of care or satisfactory relations with patients, as seen in Table 2. As health care expenditures correspond in part to doctors' incomes, this discrepant assessment of impacts is understandable.

Because the summary score was not planned initially, we did not ask what importance respondents would attribute to each of the four aspects and, thus, how much each aspect would influence their own global opinion on each tool. The summary score we computed assigns equal importance to each aspect, which is arbitrary. Other weighting schemes may lead to different results, but none would capture the inter-individual differences. Furthermore, the correlations between impacts may be partly due to a halo effect, whereby the general attitude toward a managed care tool influences the specific ratings. Importantly, correlation between the four impacts does not preclude differences between ratings in absolute terms.

### Limitations

By asking closed-format questions, we have constrained the respondents' ability to express their detailed views about the managed-care tools and to explain the reasons for their ratings. Another limitation is that we did not provide definitions of the terms employed. E.g., "health care costs" probably meant medical expenditures to most respondents, who may not have thought about indirect costs or non-medical costs. This, and similar differences in other definitions, may have added unexplained variance to the opinions.

The moderate response rate, although typical for doctor surveys, raises the issue of selection bias. Respondents may have been more critical toward managed care than non-respondents, but have no data to substantiate this belief.

We conducted this survey among a population of doctors who work within a given health system. Our results may be safer to generalize to contexts where the ethos of independent liberal practice is still present. However, more general findings may be valid regardless of context: e.g., familiar procedures may be seen as having a more positive impact than unfamiliar procedures, and tools that are imposed from the outside may be less acceptable that those that allow a measure of control by the doctors themselves. But generalisability is an empirical question, and defining the validity of our results in other contexts would require further surveys.

Regarding the issue of familiarity, we did not document each doctor's experience with each of the tools, beyond participation in a managed care organisation. In some cases, therefore, the rating reflected expectations, in others, experience. These situations are not equivalent and averaging opinions of these types of respondents may result in loss of information.

Finally, this study concerned itself only with the opinions of doctors. Other stakeholders in the health care system may have valid, yet different, opinions about the same managed care tools.

## Conclusion

Doctors in Geneva, Switzerland, expressed a positive attitude only toward the use of guidelines and otherwise held predominantly negative opinions about managed care tools. They were particularly severe concerning selective contracting, utilization review, and pay for performance. While they agreed that several measures can help control health care costs, they were particularly concerned about loss of autonomy, worsening of relations with patients, and reduced quality of care. While we did not query the respondents about their preferences, our results suggest that managed care tools and incentives that remain at least partially under control of the medical profession and that interfere the least with the current payment mechanisms may have the greatest acceptability.

From a policy perspective these generally negative attitudes are informative, as they may influence attempts at implementing managed care tools on a larger scale. Further research in this area should address the following: qualitative analysis of doctors' opinions, assessment of various combinations of managed care tools from the doctors' perspective, and assessment of the opinions of the general public regarding these policies.

## Competing interests

The authors declare that they have no competing interests.

## Authors' contributions

TP and PB proposed the study; MD, PB and TP wrote the protocol; MD conducted the survey; MD and TA analysed the data; all authors interpreted the results; MD wrote the paper and TA, PB and TP revised the paper.

## Pre-publication history

The pre-publication history for this paper can be accessed here:

http://www.biomedcentral.com/1472-6963/10/331/prepub
